# Locoregional recurrence in studies of primary systemic therapy in early invasive breast cancer

**DOI:** 10.1016/j.breast.2024.103791

**Published:** 2024-08-27

**Authors:** Luqi Chen, Stuart A. McIntosh, Siddharth Tyagi, David Dodwell

**Affiliations:** a Oxford Population Health, Richard Doll Building, Old Road Campus, University of Oxford, Oxford, OX3 7LF, UK; b The Patrick G Johnston Centre for Cancer Research, Queen's University, Belfast, BT9 7AE, UK; c King's Mill Hospital, Mansfield Rd, Sutton-in-Ashfield, NG17 4JL, UK

## Abstract

**Introduction:**

The use of primary systemic therapy (PST) in early invasive breast cancer is routine but there are concerns about risk of locoregional recurrence.

**Methods:**

We conducted a systematic literature review to identify studies of locoregional treatment and recurrence in patients with early invasive breast cancer who received non-endocrine PST.

**Results:**

We identified 112 studies (18 prospective trials and 94 non-interventional studies). The use of surgery and radiotherapy after PST was recorded in 65 (58 %) and 50 (45 %) of studies respectively. 66 (59 %) studies reported locoregional recurrence. Cumulative 5-year locoregional recurrence risks varied from 1 % to 23 %. Locoregional recurrence was higher in patients under the age of 40, those who did not achieve a pathological complete remission after PST, had ER-negative or HER2 negative tumours, were recorded to have inoperable disease before PST, and did not have radiotherapy. LRR rates in these studies have not fallen over the overall calendar period of patient enrollment (1999–2016).

**Conclusion:**

The recording of locoregional treatments and outcomes is suboptimal in studies of PST and efforts to improve this are required. In the absence of randomised evidence, our findings may help to inform care and guideline development. We were unable to exclude concern that the use of PST is associated with a higher than desired risk of locoregional recurrence.

## 1 Introduction

The use of primary systemic therapy (PST) in early invasive breast cancer prior to locoregional treatment has increased markedly in the last 2-3 decades. Initially used only in locally advanced disease, PST is now in routine use in operable early breast cancer, particularly in patients with HER2-positive or triple negative disease. PST allows downstaging of disease in the breast and axilla to allow a reduction in the extent of surgical therapy, thus reducing morbidity and allowing successful breast conserving therapy (BCT) in many patients, with its associated improvements in quality of life and patient satisfaction compared to mastectomy [1,2]. In addition, response to PST commonly determines the use of effective post-surgical therapy and may facilitate more rapid progress in clinical research.

In 2018 the Early Breast Cancer Trialists’ Collaborative Group (EBCTCG) published an individual patient level meta-analysis of 4756 patients in 10 randomized trials, which began before 2005 of neoadjuvant vs adjuvant chemotherapy. Distant recurrence and survival were unaffected by the sequencing of surgery and chemotherapy, but locoregional recurrence was significantly higher in those patients who received neoadjuvant chemotherapy [3]. Exploration of the cause of this worrying observation was hindered by the lack of detail concerning the type of locoregional treatment undertaken at individual patient level and the limited available surgical and pathological information. The implications of this finding for current care are therefore unclear but obviously raise concerns that the reduced extent of surgery may be responsible. This may act as a barrier to the appropriate use of PST in some circumstances.

We conducted a systematic literature review to explore the recording of locoregional recurrence in studies of non-endocrine PST in early invasive breast cancer.

## 2 Methods

### 2.1 Search methods

**Fig. 1:**
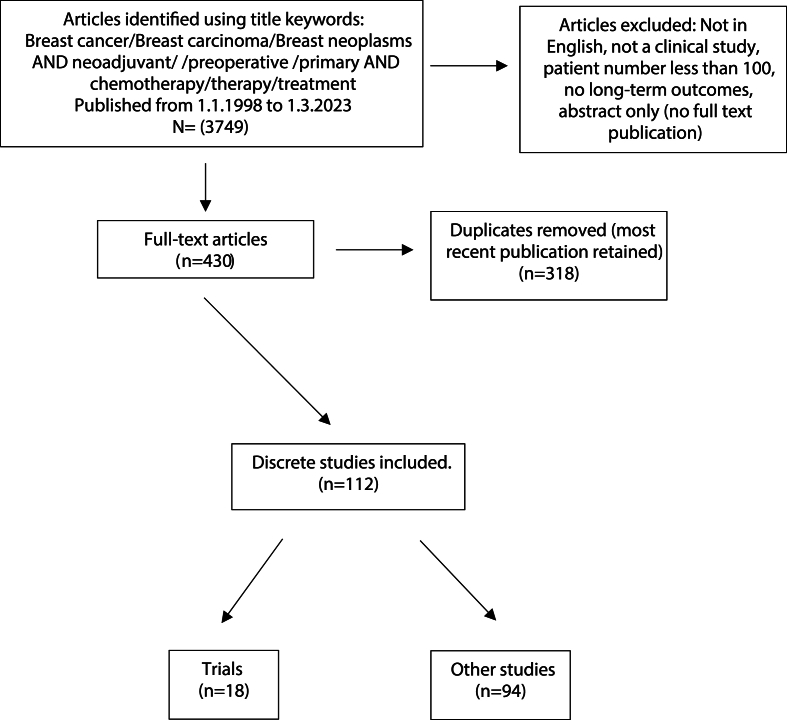
Consort Diagram.

A systematic search was conducted through the online databases PubMed, clinicaltrial.gov.com, and the Cochrane Library for articles in English, published between January 1, 1998 and March 1, 2023 to identify studies that describe survival and recurrence outcomes in patients with early invasive breast cancer who underwent PST. The search used the following non-restrictive keywords: Breast cancer/Breast carcinoma/Breast neoplasms AND Neoadjuvant/Preoperative/Primary AND Cytotoxic/Chemotherapy/Therapy/Treatment. One researcher (LC) reviewed the title and abstract of all publications identified to exclude those that did not address the research question. LC and DD resolved uncertainties. Remaining full text publications were further reviewed and non-applicable articles removed (Fig. 1). Clinical trials and non-interventional (observational) studies were included. We included publications describing studies of ≥100 patients that reported any long-term (≥5 year) outcomes for recurrence and/or death. For studies also reporting the outcomes for patients undergoing post-operative systemic therapy without PST, data were extracted for patients who received PST alone. Studies reporting on primary, pre-operative or neo-adjuvant endocrine therapy were not included. Abstracts without full publications and duplicates were removed.

The systematic review was conducted according to guidance from the Cochrane organization [4] and was prospectively registered in PROSPERO (2022-CRD42022331483) [5]. We extracted data on study type, year of publication, recruitment period, patient and tumour characteristics, treatment, and outcomes (Supplementary Table 1).

### 2.2 Statistical analysis

We analyzed the association between 5-year cumulative locoregional recurrence risk (LRR) and the following variables: type and size of study, year of completion of recruitment, use of breast conserving surgery, radiotherapy, endocrine therapy, HER2 targeting therapy, postoperative chemotherapy and the achievement of pathological complete response (pCR). LRR was as defined by study authors but, where specified, did not include contralateral events. We describe associations for studies that reported 5-year LRR risks as these were the majority. We also calculated an annual LRR rate to consider any associations between LRR rate and study size, type and calendar period of recruitment. Linear regression analysis was used to ascertain the correlation of different data groups. For the meta-analysis, summary data were synthesized with a random effects model to ascertain the risk of LRR when the I^2^ is more than 50 %, and fixed effects model if I^2^ is less than 50 %. Odds ratios were utilized to identify the association between different risk factors and LRR, Risk ratios were used to identify the effect of RT. A two-sided p value of less than 0.05 was considered to be statistically significant. Calculations were performed using SPSS version 23.0 (IBM Corp., Armonk, USA). Figures were created in Review Manager 5.3 and BioRender. Meta-analyses were conducted using Review Manager (RevMan version).

## 3 Results

A total of 112 publications relating to discrete studies were identified. These included 18 clinical trials and 94 non-interventional studies (Fig. 1, Supplementary Table 1).

Recruitment periods spanned 1997 to 2021 and 1997 to 2022 for trials and non-interventional studies respectively. The reporting of study type and size, patient and tumour characteristics, treatment and outcomes is provided in Supplementary Table 1.

### 3.1 Recording of locoregional therapy

Final breast surgery (breast conserving therapy or mastectomy) was recorded in 77 % and 73 % of trials and non-interventional studies respectively, similar to the reporting of axillary surgery (71 % and 69 %). The use of post-operative radiotherapy was poorly recorded (17 % and 51 %) respectively with no improvement over calendar period.

### 3.2 Locoregional recurrence

Fourteen trials (78 %) and 52 non-interventional studies (55 %) reported locoregional recurrence risk (LRR) separately from any other outcome reporting. Five-year cumulative LRR risks varied from 2 % to 12.4 % in clinical trials and from 1 to 21.3 % in non-interventional studies. LRR was not the primary endpoint of any study.

A reduced risk of LRR was seen in patients whose age was ≥40 at diagnosis (OR 0.63 [95 % confidence interval 0.44–0.91] – Fig. 2a), those who achieved pathological complete response–pCR (OR 0.36 [0.20–0.67] Fig. 2b) and who add had ER + disease (OR 0.65 [0.44–0.95] Fig. 2c). There was no statistically significant difference according to type of breast surgery (mastectomy vs BCS, Fig. 2d).

**Fig. 2a:**
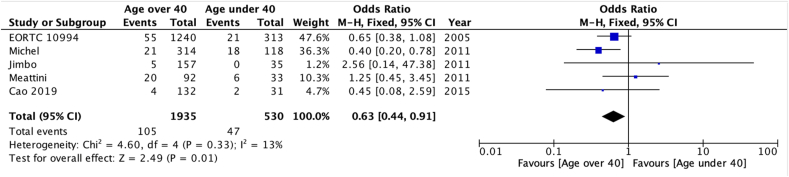
Odds ratios and 95 % confidence Intervals for locoregional recurrence by age at diagnosis (<40 vs 40+ years)

**Fig. 2b:**
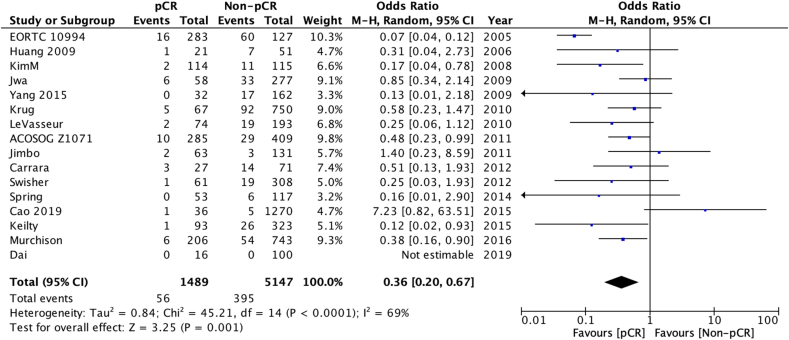
Odds ratios and 95 % confidence intervals for locoregional recurrence by achievement of pathological complete response (pCR)

**Fig. 2c:**
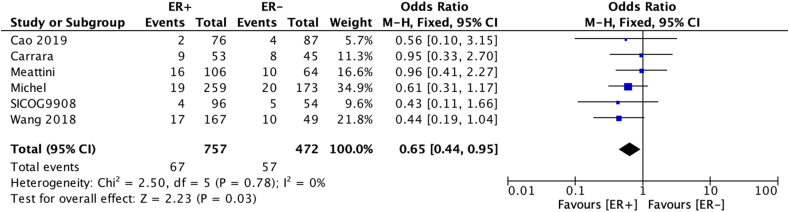
Odds ratios and 95 % confidence intervals for locoregional recurrence by ER status

**Fig. 2d:**
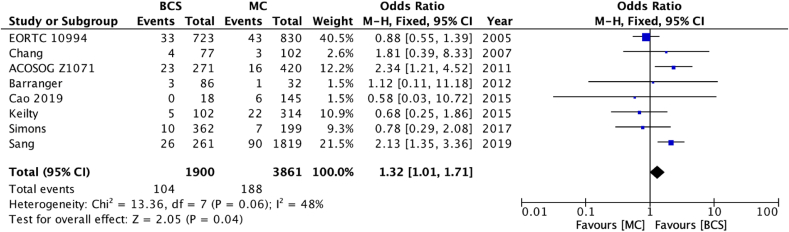
Odds ratios and 95 % confidence intervals for locoregional recurrence by surgery (BCS = breast **conserving surgery, MC=mastectomy)**.

Patients with HER2 negative disease (OR 0.57 [0.47–0.68] Fig. 3a), who had operable disease at diagnosis (OR 0.43 [0.27–0.71] Fig. 3b), received post-operative radiotherapy (OR 0.55 [95 % confidence interval 0.37–0.82] – Fig. 3c) also had a lower risk of LRR. There was no statistically significant difference in LRR according to use of post-operative chemotherapy (data not shown).

**Fig. 3a:**
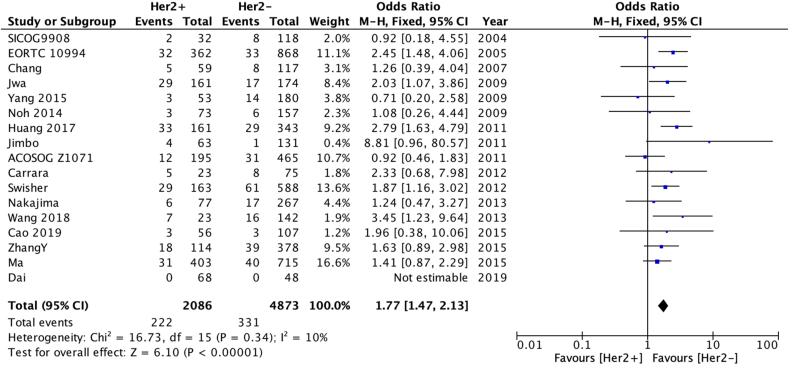
Odds ratios and 95 % confidence intervals for locoregional recurrence by HER2 status.

**Fig. 3b:**
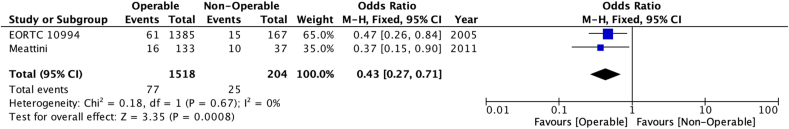
Odds ratios and 95 % confidence **intervals for locoregional recurrence by operability of disease assessed at diagnosis**

**Fig. 3c:**
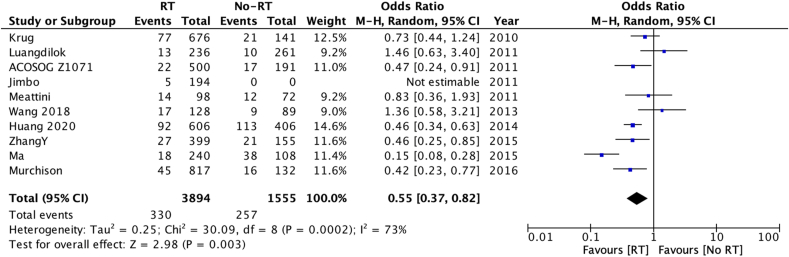
Odds ratios and 95 % confidence intervals for locoregional recurrence by use of radiotherapy.

**Fig. 4:**
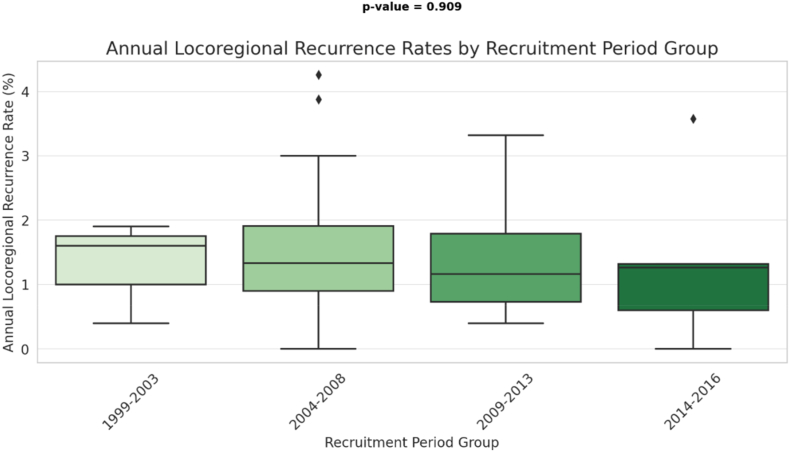
Annual locoregional recurrence rate by recruitment period.

Calculated annual rates of LRR (to allow inclusion of all studies reporting cumulative risk of LRR at any time point) varied from 0.4 to 2.5 % and from 0 to 4.3 % in clinical trials and non-interventional studies respectively. LRR rates were somewhat higher in medium size (300–700 patients) studies than in those that were smaller or larger (supplementary Figure 1a) but were unaffected by study type (supplementary Figure 1b) or calendar period of recruitment (Fig. 4).

## 4 Discussion

The contemporary relevance of higher-than-expected LRR following the use of primary systemic therapy, compared to post-operative adjuvant therapy, reported in the EBCTCG meta-analysis [3] and the Cochrane review [6] is disputed [[7], [8], [9], [10]]. It is unlikely that further randomized trials of PST vs the same therapy given post-operatively will ever be performed, so there are limited opportunities to be certain that there is no excess risk of LRR following the use of PST in contemporary care. The main motivation for this study was to assess the reporting of LRR following PST in more recently published studies.

We identified a relative lack of recording of locoregional treatments and locoregional recurrence, even in studies conducted during the last decade. Although systemic therapies were well recorded in the trials and the non-interventional studies of PST that we identified, details of surgery to both the breast and axilla were less well, but equally, recorded in both study categories. Radiotherapy was surprisingly, particularly poorly recorded in prospective trials. Locoregional recurrence was also not recorded as a discrete event in 41 % of studies, although may have been included within a composite endpoint in some.

Indirect comparisons between study types, particularly given the incomplete recording of LRR at study level, require cautious interpretation, but risks of LRR comparing results from prospective trials and non-interventional studies appear comparable.

Our study has strengths. We conducted a literature review over a thirty-year period with non-restrictive search terms necessitating extensive manual searching of studies of PST reporting recurrence and mortality at a minimum follow-up of 5 years, with a focus on locoregional treatment and outcomes. We used data on LRR at 5 years and calculated annual rate of LRR for some analyses.

Weaknesses include a reliance on published literature, limiting the number of studies included in the analyses of factors that may have contributed to LRR, and that we did not perform an individual patient level meta-analysis. This was not possible given the large number of non-interventional studies included and the availability of data, but limited the depth of analyses and in particular, a greater exploration of confounding that may have explained some of the associations between study, patient and tumour characteristics, and treatment, with LRR. A specific limitation was the inability to consider the use of surgery and radiotherapy at patient level. A further issue, common to all analyses of mature studies in early breast cancer is their relevance to contemporary care, given the substantial changes in systemic and local treatments and the improvements in outcomes seen over recent decades. The lack of any major changes in the recording of locoregional treatments and outcomes over the ∼30-year period of accrual of patients in these studies, suggests that these results remain relevant to current care.

Two recent influential meta-analyses of the use of PST reported on overall recurrence but did not address LRR separately [11,12]. However, a pooled analysis of 9 prospective breast cancer trials did report that positive predictors of locoregional recurrence after PST, included young age, positive clinical nodal status, higher tumour grade, HER2+ or triple negative disease subtype, and non-achievement of pCR [13]. Our findings are consistent with this study. In the absence of randomized evidence to guide locoregional management, particularly radiotherapy after PST, these findings may be useful in informing care.

## 5 Conclusions

PST is a major treatment advance in the management of early breast cancer but concerns about LRR risk and its link with increased long-term breast cancer mortality remain. More comprehensive reporting of locoregional treatments and recurrence in ongoing and future trials of PST are required to address these concerns and represent the most pragmatic means to provide improved confidence in the increasing use of PST in modern care. The PRECEDENT project was set up recently to establish a core outcome set and reporting requirements for locoregional therapy in trials of PST. Their uniform adoption would achieve this goal [14].

## Funding

Funding was provided by 10.13039/501100000289Cancer Research UK (grant C7852–A25447) and the 10.13039/501100000769University of Oxford. These funding bodies had no role in the data collection, analysis, interpretation, or reporting.

## Competing interests

10.13039/100014670SM reports speaker honoraria from 10.13039/100030732MSD, 10.13039/100004337Roche, 10.13039/100017412BD and Astra Zeneca; advisory boards for Lilly, 10.13039/100004336Novartis, 10.13039/100030732MSD, 10.13039/100004337Roche and Astra Zeneca; conference travel and support from 10.13039/100004337Roche, Lilly, 10.13039/100004336Novartis and 10.13039/100030732MSD. DD reports honoraria from Pfizer. All authors have completed the ICMJE uniform disclosure form at www.icmje.org/coi_disclosure.pdf and declare: no support from any organisation for the submitted work other than salaries from their employing institutions and the funding listed in the section above; no financial relationships with any organisations that might have an interest in the submitted work in the previous three years; and no other relationships or activities that could appear to have influenced the submitted work.

## Patient input

Two patient members of ICPV (https://www.independentcancerpatientsvoice.org.uk) reviewed and suggested amendments to the manuscript and will help with dissemination of the findings.

## Ethical approval

Ethical approval was not required.

## Data sharing

Extensive source information and publication references are provided.

## Transparency

The corresponding author (DD) affirms that this manuscript is an honest, accurate, and transparent account of the study being reported; that no important aspects of the study have been omitted; and that any discrepancies from the study as planned have been explained.

## Open access

This is an open access article distributed in accordance with the terms of the Creative Commons Attribution (CC BY 4.0) license, which permits others to distribute, remix, adapt and build upon this work, for commercial use, provided the original work is properly cited. See: http://creativecommons.org/licenses/by/4.0. This article has not been deposited as a preprint.

## Copyright statement

The Corresponding Author has the right to grant on behalf of all authors and does grant on behalf of all authors, a worldwide license to the Publishers and its licensees in perpetuity, in all forms, formats and media (whether known now or created in the future), to i) publish, reproduce, distribute, display and store the Contribution, ii) translate the Contribution into other languages, create adaptations, reprints, include within collections and create summaries, extracts and/or, abstracts of the Contribution, iii) create any other derivative work(s) based on the Contribution, iv) to exploit all subsidiary rights in the Contribution, v) the inclusion of electronic links from the Contribution to third party material where-ever it may be located; and, vi) license any third party to do any or all of the above.

## CRediT authorship contribution statement

**Luqi Chen:** Writing – review & editing, Writing – original draft, Validation, Methodology, Investigation, Formal analysis, Data curation, Conceptualization. **Stuart A. McIntosh:** Writing – review & editing, Validation, Methodology, Investigation. **Siddharth Tyagi:** Writing – review & editing, Conceptualization. **David Dodwell:** Writing – review & editing, Writing – original draft, Validation, Supervision, Project administration, Methodology, Funding acquisition, Conceptualization.
