# Supplementary material for: Locoregional recurrence in studies of primary systemic therapy in early invasive breast cancer
Source: Breast. 2024 Aug 27;77:103791. doi: 10.1016/j.breast.2024.103791 (PMC11402831; doi:10.1016/j.breast.2024.103791)
Supplement: Multimedia component 1 [file mmc1.docx]

**a)**

**
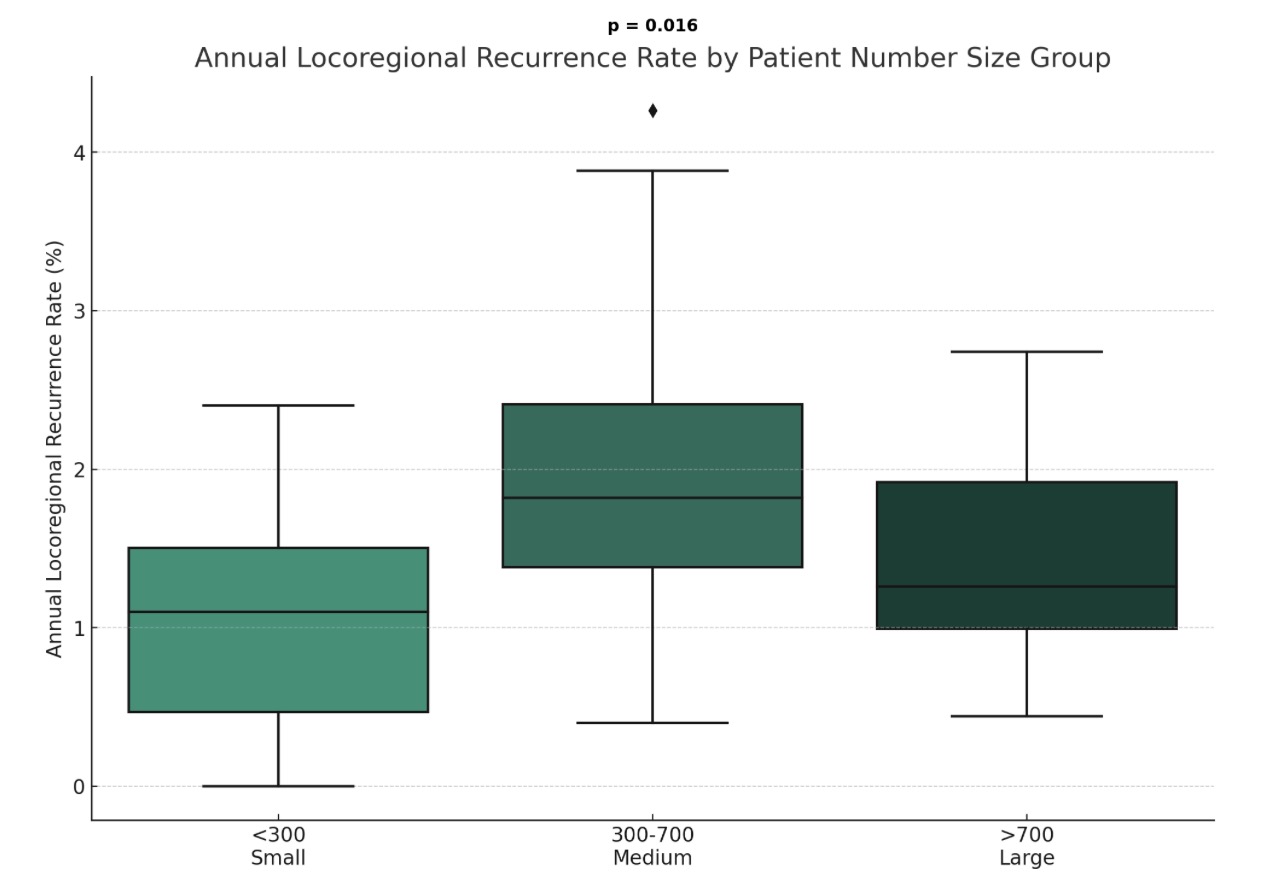
**

**b)**

**
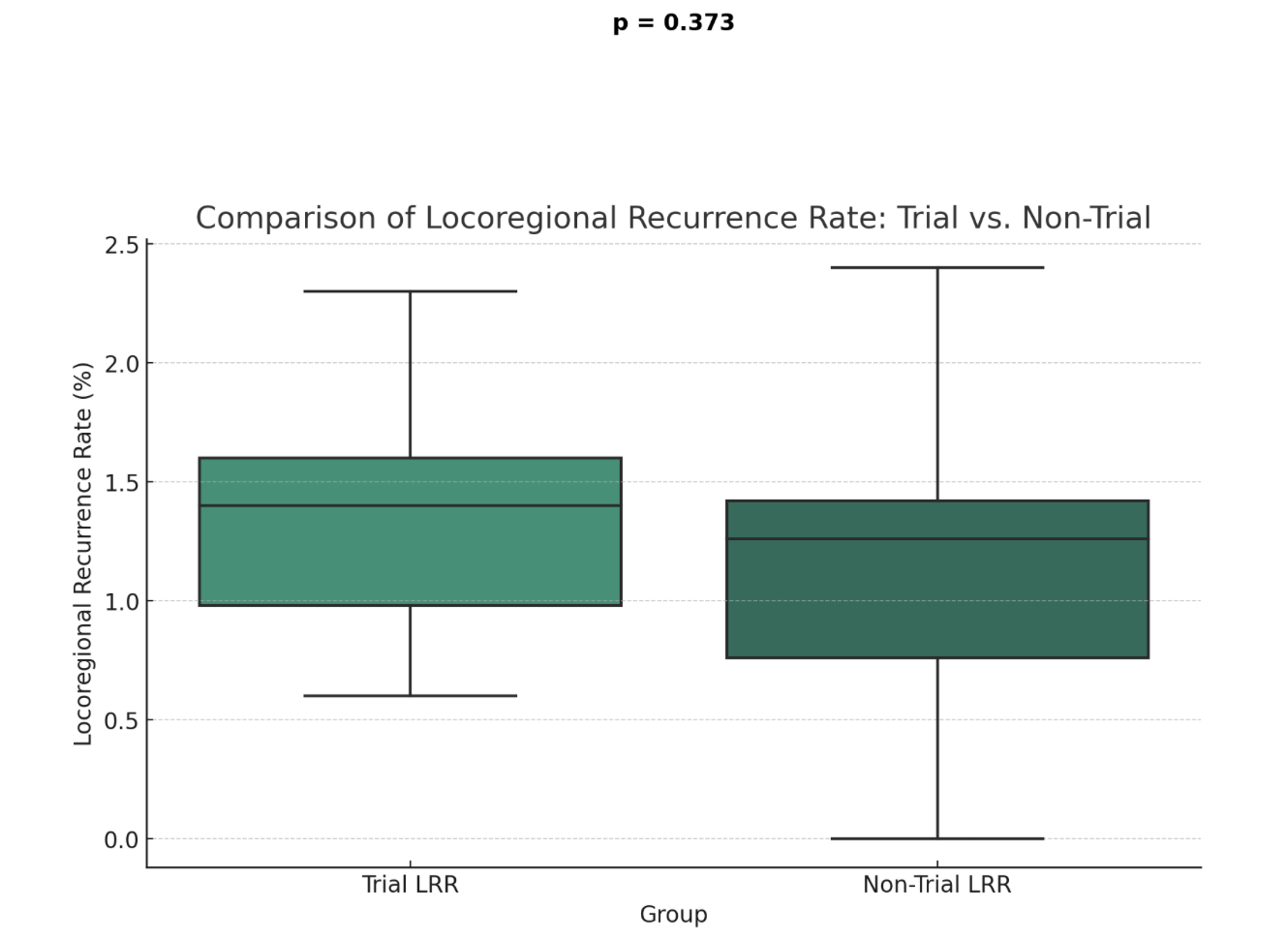
**

**Supplementary Figure 1 Annual locoregional recurrence rate by study size and type**

**Supplementary Table 1 - Included studies and references**

**Observational studies**

|  | Trial/study name | Recruitment period | Country | No. of centres | Median months of  Follow-up | Median/Mean age (range) | No. of patients | Operability at diagnosis | HER2 positive | ER positive | Chemotherapy category | % who had BCS | Post-operative chemotherapy recorded | Radiotherapy recorded | Total pCR | Overall Survival (years) | Disease-free survival  (years) | Locoregional recurrence (years) | Annual Locoregional recurrence% |
| --- | --- | --- | --- | --- | --- | --- | --- | --- | --- | --- | --- | --- | --- | --- | --- | --- | --- | --- | --- |
| **[1]** | **Arun** | **1997-2009** | **NA** | **NA** | **38** | **NA** | **317** | **86%** | **19%** | **62%** | **NA** | **19%** | **NA** | **NA** | **26.00%** | **81.0% (5)** | **NA** | **NA** | **NA** |
| **[2]** | **Miller** | **1997-2010** | **NA** | **NA** | **31** | **49 (26-79)** | **218** | **88%** | **30%** | **NA** | **5** | **NA** | **NA** | **NA** | **27%** | **75.4% (5)** | **NA** | **NA** | **NA** |
| **[3]** | **Meattini** | **1997-2011** | **Italy** | **1** | **NA** | **49 (24-76)** | **170** | **39%** | **NA** | **39%** | **NA** | **NA** | **14%** | **0%** | **NA** | **57.6% (5)** | **NA** | **7.1% (5)** | **1.42%** |
| **[3]** | **Meattini** | **1997-2011** | **Italy** | **1** | **NA** | **49 (24-76)** | **170** | **39%** | **NA** | **39%** | **NA** | **NA** | **6%** | **100%** | **NA** | **NA** | **NA** | **7.6% (5)** | **1.42%** |
| **[4]** | **Nakhlis** | **1997-2014** | **USA** | **1** | **44** | **50 (28-89)** | **181** | **NA** | **38%** | **NA** | **NA** | **NA** | **33%** | **100%** | **21%** | **56.3% (5)** | **NA** | **1.1% (5)** | **0.22%** |
| **[5]** | **Noh 2014** | **1998-2009** | **Korea** | **NA** | **66** | **NA** | **260** | **NA** | **NA** | **NA** | **NA** | **100%** | **NA** | **100%** | **NA** | **96.4% (5)** | **90.5% (5)** | **4.5% (5)** | **0.76%** |
| **[6]** | **Spring** | **1998-2014** | **USA** | **1** | **77** | **NA** | **170** | **NA** | **32%** | **NA** | **NA** | **38%** | **32%** | **82%** | **31%** | **81.2% (5)** | **63.6% (5)** | **3.5% (5)** | **0.70%** |
| **[7]** | **Huang 2017** | **1999-2011** | **China** | **1** | **61** | **49 (24-75)** | **510** | **85%** | **NA** | **NA** | **NA** | **NA** | **NA** | **NA** | **0%** | **79.0% (5)** | **63.1% (5)** | **12.2% (5)** | **0.90%** |
| **[8]** | **Ma** | **1999-2015** | **China** | **1** | **89** | **NA** | **1118** | **77%** | **36%** | **60%** | **NA** | **NA** | **92%** | **63%** | **NA** | **NA** | **NA** | **6.3% (5)** | **1.26%** |
| **[9]** | **Alvarado-Miranda** | **2000-2003** | **Mexico** | **1** | **43** | **50 (39-61)** | **112** | **64%** | **NA** | **43%** | **5** | **0%** | **NA** | **100%** | **30%** | **84.2% (5)** | **76.9% (5)** | **NA** | **NA** |
| **[10]** | **Kobayashi** | **2000-2007** | **Japan** | **1** | **82** | **51 (25-75)** | **258** | **75%** | **NA** | **NA** | **NA** | **NA** | **NA** | **NA** | **NA** | **82.7% (5)** | **NA** | **NA** | **NA** |
| **[11]** | **Yang 2015** | **2000-2009** | **NA** | **NA** | **62** | **46 (24-81)** | **233** | **NA** | **23%** | **NA** | **5** | **NA** | **NA** | **100%** | **14%** | **79.0% (5)** | **69% (5)** | **8.0% (5)** | **1.60%** |
| **[12]** | **Chen 2012** | **2000-2008** | **china** | **1** | **60** | **NA** | **224** | **89%** | **21%** | **84%** | **5** | **0%** | **NA** | **86%** | **14%** | **79.8% (5)** | **65.1% (5)** | **NA** | **NA** |
| **[13]** | **Huang 2020** | **2000-2014** | **China** | **NA** | **73** | **49 (20-79)** | **1813** | **NA** | **31%** | **67%** | **NA** | **0%** | **NA** | **70%** | **NA** | **84.0% (5)** | **83.1% (5)** | **13.7% (5)** | **2.74%** |
| **[14]** | **Terman** | **2000-2020** | **USA** | **1** | **81** | **NA** | **2196** | **NA** | **13%** | **NA** | **NA** | **NA** | **NA** | **NA** | **NA** | **87.3% (5)** | **77.3% (5)** | **NA** | **NA** |
| **[15]** | **Huang 2009** | **2001-2006** | **china** | **1** | **63** | **52 (25-70)** | **119** | **NA** | **28%** | **51%** | **3** | **0%** | **100%** | **100%** | **19%** | **71.3% (5)** | **58.7% (5)** | **1.7% (5)** | **0.34%** |
| **[16]** | **Lee 2012** | **2001-2006** | **Korea** | **1** | **71** | **NA** | **813** | **NA** | **22%** | **68%** | **NA** | **60%** | **87** | **96%** | **NA** | **NA** | **73.0% (5)** | **NA** | **NA** |
| **[17]** | **Min** | **2001-2006** | **NA** | **NA** | **NA** | **NA** | **251** | **NA** | **NA** | **NA** | **NA** | **NA** | **NA** | **NA** | **13%** | **NA** | **10.8% (5)** | **NA** | **NA** |
| **[18]** | **KimM** | **2001-2008** | **USA** | **NA** | **63** | **NA** | **229** | **87%** | **NA** | **40%** | **NA** | **41%** | **NA** | **NA** | **100%** | **85.0% (5)** | **NA** | **0.0% (5)** | **0.00%** |
| **[19]** | **Noh 2015** | **2001-2009** | **Korea** | **NA** | **57** | **50** | **110** | **NA** | **14%** | **NA** | **NA** | **36%** | **NA** | **NA** | **NA** | **75.5% (5)** | **60.2% (5)** | **3.6% (5)** | **0.90%** |
| **[20]** | **Takada** | **2001-2010** | **Japan** | **NA** | **42** | **53 (25-70)** | **776** | **100%** | **100%** | **NA** | **NA** | **NA** | **NA** | **NA** | **52%** | **NA** | **84.8% (5)** | **NA** | **NA** |
| **[21]** | **Garc ́ıa-Torralba** | **2001-2010** | **Spain** | **1** | **144** | **56 (21-79)** | **121** | **NA** | **24%** | **NA** | **2** | **44%** | **NA** | **88%** | **17%** | **75.8% (10)** | **73.3% (5)** | **NA** | **NA** |
| **[22]** | **Murchison** | **2001-2016** | **Canada** | **NA** | **68** | **NA** | **949** | **78%** | **35%** | **39%** | **NA** | **22%** | **NA** | **100%** | **21%** | **NA** | **NA** | **5.0% (5)** | **1.00%** |
| **[23]** | **Chang** | **2002-2007** | **Korea** | **1** | **61** | **46 (28-69)** | **179** | **NA** | **32%** | **NA** | **2** | **43%** | **NA** | **88%** | **8%** | **89.9% (5)** | **74.9% (5)** | **7.3% (5)** | **1.46%** |

|  | Trial/study name | Recruitment period | country | centres | Follow-up (Median month | Median/Mean age (Range) | No. of patients | Operability at diagnosis | HER2 positive (%) | ER positive (%) | Chemotherapy Category* | BCS Surgery pts | Post-operative chemotherapy recorded | Radiotherapy recorded | Total pCR | Overall Survival (years) | Disease-free survival  (years) | Locoregional recurrence (years) | Annual Locoregional recurrence% |
| --- | --- | --- | --- | --- | --- | --- | --- | --- | --- | --- | --- | --- | --- | --- | --- | --- | --- | --- | --- |
| **[24]** | **Jwa** | **2002-2009** | **Korea** | **1** | **86** | **48 (28-76)** | **335** | **NA** | **NA** | **NA** | **NA** | **NA** | **NA** | **NA** | **11%** | **NA** | **NA** | **3.5% (5)** | **0.70%** |
| **[24]** | **Jwa** | **2002-2009** | **Korea** | **1** | **86** | **48 (28-76)** | **335** | **NA** | **NA** | **NA** | **NA** | **NA** | **NA** | **NA** | **6%** | **NA** | **NA** | **9.1% (5)** | **1.82%** |
| **[24]** | **Jwa** | **2002-2009** | **Korea** | **1** | **86** | **48 (28-76)** | **335** | **NA** | **NA** | **NA** | **NA** | **NA** | **NA** | **NA** | **19%** | **NA** | **NA** | **12.0% (5)** | **2.40%** |
| **[24]** | **Jwa** | **2002-2009** | **Korea** | **1** | **86** | **48 (28-76)** | **335** | **NA** | **NA** | **NA** | **NA** | **NA** | **NA** | **NA** | **21%** | **NA** | **NA** | **7.1% (5)** | **1.42%** |
| **[24]** | **Jwa** | **2002-2009** | **Korea** | **1** | **86** | **48 (28-76)** | **335** | **NA** | **NA** | **NA** | **NA** | **NA** | **NA** | **NA** | **36%** | **NA** | **NA** | **19.4% (5)** | **3.88%** |
| **[24]** | **Jwa** | **2002-2009** | **Korea** | **1** | **86** | **48 (28-76)** | **335** | **NA** | **NA** | **NA** | **NA** | **NA** | **NA** | **NA** | **23%** | **NA** | **NA** | **21.3% (5)** | **4.26%** |
| **[25]** | **Wong** | **2002-2014** | **USA** | **1** | **NA** | **NA** | **967** | **NA** | **NA** | **NA** | **NA** | **NA** | **NA** | **NA** | **NA** | **85.5% (5)** | **60.4% (5)** | **11.7% (5)** | **2.34%** |
| **[26]** | **Michel** | **2003-2011** | **German** | **NA** | **59** | **48 (20-74)** | **432** | **94%** | **24%** | **60%** | **NA** | **78%** | **NA** | **NA** | **29%** | **NA** | **NA** | **10% (5)** | **2.00%** |
| **[27]** | **Dredze** | **2003-2012** | **Isreal** | **1** | **NA** | **49 (23-72)** | **200** | **92%** | **33%** | **67%** | **NA** | **49%** | **NA** | **99%** | **26%** | **60.0% (10)** | **NA** | **1.0% (10)** | **0.10%** |
| **[28]** | **Chen 2019** | **2003-2012** | **China** | **1** | **75** | **50 (25-73)** | **353** | **NA** | **21%** | **73%** | **NA** | **NA** | **NA** | **NA** | **13%** | **NA** | **74.8% (5)** | **NA** | **NA** |
| **[29]** | **Zhang J** | **2003-2015** | **China** | **1** | **81** | **48 (23-73)** | **1585** | **100%** | **41%** | **59%** | **NA** | **40%** | **NA** | **NA** | **24%** | **90.0% (5)** | **NA** | **NA** | **NA** |
| **[30]** | **Kwon** | **2003-2015** | **Korea** | **NA** | **101** | **46 (40-54)** | **318** | **NA** | **NA** | **NA** | **2** | **54%** | **52%** | **91%** | **5%** | **97.0% (5)** | **NA** | **NA** | **NA** |
| **[31]** | **Shin** | **2004-2007** | **korea** | **NA** | **67** | **46 (37-54)** | **166** | **80%** | **31%** | **53%** | **5** | **44%** | **NA** | **NA** | **NA** | **NA** | **NA** | **NA** | **NA** |
| **[32]** | **Kim 2010** | **2004-2008** | **Korea** | **1** | **23** | **NA** | **257** | **NA** | **30%** | **NA** | **2** | **16%** | **100%** | **100%** | **10%** | **NA** | **80.1% (5)** | **NA** | **NA** |
| **[33]** | **Jimbo** | **2004-2011** | **Japan** | **1** | **61** | **NA** | **363** | **NA** | **29%** | **NA** | **NA** | **NA** | **NA** | **92%** | **NA** | **NA** | **NA** | **6.1% (5)** | **1.22%** |
| **[34]** | **Ohri** | **2004-2013** | **USA** | **NA** | **NA** | **51 (19-90)** | **29270** | **NA** | **NA** | **NA** | **NA** | **NA** | **NA** | **63%** | **NA** | **80.0% (5)** | **NA** | **NA** | **NA** |
| **[35]** | **Haque** | **2004-2014** | **USA** | **NA** | **NA** | **NA** | **13939** | **82%** | **NA** | **NA** | **NA** | **37%** | **NA** | **NA** | **19%** | **57.1% (5)** | **NA** | **NA** | **NA** |
| **[36]** | **Prakash** | **2004-2014** | **USA** | **NA** | **58** | **51 (43-59)** | **85143** | **NA** | **32%** | **NA** | **NA** | **37%** | **NA** | **75%** | **NA** | **80.0% (5)** | **NA** | **NA** | **NA** |
| **[37]** | **van Nijnatten** | **2005-2008** | **Netherlands** | **NA** | **60** | **NA** | **8176** | **NA** | **43%** | **38%** | **NA** | **21%** | **NA** | **81%** | **NA** | **NA** | **70.1% (5)** | **NA** | **NA** |
| **[38]** | **LeVasseur** | **2005-2010** | **Canada** | **NA** | **90** | **NA** | **267** | **NA** | **34%** | **64%** | **NA** | **NA** | **NA** | **NA** | **28%** | **77.2% (5)** | **NA** | **7.8% (5)** | **1.56%** |
| **[39]** | **Swisher** | **2005-2012** | **NA** | **NA** | **55** | **NA** | **751** | **100%** | **NA** | **NA** | **NA** | **100%** | **NA** | **NA** | **32%** | **NA** | **NA** | **4.5% (5)** | **0.90%** |
| **[40]** | **Carrara** | **2005-2012** | **Brazil** | **1** | **73** | **48 (37-59)** | **449** | **NA** | **35%** | **NA** | **2** | **22%** | **NA** | **NA** | **17%** | **81.2% (5)** | **NA** | **15.0% (5)** | **3.00%** |
| **[41]** | **Nakajima** | **2005-2013** | **Japan** | **NA** | **81** | **49 (25-80)** | **351** | **76%** | **23%** | **74%** | **NA** | **0%** | **NA** | **100%** | **5%** | **83.4% (5)** | **69.8% (5)** | **8.7% (5)** | **1.74%** |
| **[42]** | **Luo** | **2005-2013** | **China** | **1** | **64** | **49 (21-80)** | **497** | **NA** | **21%** | **69%** | **NA** | **NA** | **100%** | **100%** | **NA** | **86.6% (5)** | **70.4% (5)** | **2.4% (5)** | **0.48%** |
| **[43]** | **Al-Tweigeri** | **2005-2014** | **Saudi Arabia** | **1** | **61** | **44 (38-60)** | **681** | **62%** | **36%** | **59%** | **NA** | **12%** | **NA** | **100%** | **23%** | **86.9% (5)** | **72.3% (5)** | **6.8% (5)** | **1.36%** |
| [44] | **antolín** | **2005-2016** | **Spain** | **1** | **NA** | **49 (30-79)** | **142** | **89%** | **NA** | **NA** | **NA** | **66%** | **NA** | **55%** | **52%** | **85.0% (5)** | **78.0% (5)** | **NA** | **NA** |

|  | Trial/study name | Recruitment period | country | centres | Follow-up (Median month) | Median/Mean age (Range) | No. of patients | Operability at diagnosis | HER2 positive | ER positive | Chemotherapy category | BCS Surgery pts | Post-operative chemotherapy recorded | Radiotherapy recorded | Total pCR | Overall Survival (years) | Disease-free survival (years) | Locoregional recurrence (years) | Annual Locoregional recurrence% |
| --- | --- | --- | --- | --- | --- | --- | --- | --- | --- | --- | --- | --- | --- | --- | --- | --- | --- | --- | --- |
| **[45]** | **Chen 2021** | **2005-2017** | **China** | **1** | **45** | **49** | **968** | **78%** | **45%** | **60%** | **NA** | **39%** | **74%** | **NA** | **22%** | **89.5% (5)** | **70.5% (5)** | **NA** | **NA** |
| **[46]** | **Hannikainen** | **2005-2022** | **Finland** | **1** | **42** | **54 (22-81)** | **119** | **NA** | **100%** | **NA** |  | **34%** | **98%** | **97%** | **52%** | **86.7% (5)** | **78.0% (5)** | **NA** | **NA** |
| **[47]** | **Fujita** | **2006-2018** | **Japan** | **1** | **61** | **NA** | **142** | **NA** | **NA** | **NA** | **NA** | **NA** | **NA** | **NA** | **NA** | **100.0% (5)** | **NA** | **NA** | **NA** |
| **[48]** | **Xin** | **2006-2009** | **China** | **NA** | **57** | **50 (23-72)** | **289** | **NA** | **NA** | **NA** | **NA** | **NA** | **NA** | **NA** | **NA** | **81.1% (5)** | **67.2% (5)** | **NA** | **NA** |
| **[49]** | **KimH 2021** | **2006-2015** | **Korea** | **1** | **57** | **47 (26-75)** | **223** | **91%** | **29%** | **NA** | **NA** | **22%** | **NA** | **98%** | **100%** | **92.8% (5)** | **84.7% (5)** | **5.8% (5)** | **1.16%** |
| **[50]** | **Luangdilok** | **2007-2011** | **Thiland** | **1** | **54** | **48 (24-97)** | **179** | **49%** | **40%** | **58%** | **5** | **10%** | **100%** | **94%** | **NA** | **57.0% (5)** | **56.0% (5)** | **11% (5)** | **2.20%** |
| **[51]** | **Barranger** | **2007-2012** | **France** | **1** | **41** | **50 (27-76)** | **168** | **NA** | **31%** | **NA** | **5** | **72%** | **NA** | **NA** | **NA** | **77.0% (5)** | **69.8% (5)** | **3.4% (5)** | **0.68%** |
| **[52]** | **Zhu** | **2007-2014** | **China** | **NA** | **57** | **50 (22-81)** | **233** | **NA** | **65%** | **46%** | **NA** | **NA** | **NA** | **NA** | **10%** | **93.3% (5)** | **NA** | **5.2% (5)** | **1.04%** |
| **[53]** | **ZhangY** | **2007-2015** | **China** | **1** | **65** | **51 (22-78)** | **554** | **NA** | **61%** | **21%** | **NA** | **0%** | **NA** | **72%** | **38%** | **NA** | **74.2% (5)** | **9.2 (5)** | **1.84%** |
| **[54]** | **Munoz-Montano** | **2007-2015** | **Mexico** | **1** | **53** | **49 (22-88)** | **1519** | **NA** | **NA** | **NA** | **NA** | **NA** | **NA** | **87%** | **33%** | **91.9% (5)** | **77.4% (5)** | **NA** | **NA** |
| **[55]** | **Asaoka** | **2007-2016** | **Japan** | **NA** | **63** | **NA** | **394** | **NA** | **54%** | **NA** | **2** | **NA** | **NA** | **57%** | **100%** | **NA** | **92.3% (5)** | **2% (5)** | **0.40%** |
| **[56]** | **Woo** | **2007-2016** | **Korea** | **1** | **48** | **45 (22-75)** | **1017** | **100%** | **33%** | **NA** | **NA** | **59%** | **NA** | **NA** | **NA** | **72.5% (5)** | **NA** | **NA** | **NA** |
| **[57]** | **Chun** | **2007-2017** | **Korea** | **NA** | **59** | **47 (24-80)** | **676** | **97%** | **33%** | **NA** | **NA** | **43%** | **NA** | **17%** | **NA** | **98.0% (5)** | **NA** | **NA** | **NA** |
| **[58]** | **Gwark** | **2008-2014** | **Korea** | **1** | **66** | **46 (36-56)** | **1641** | **94%** | **32%** | **NA** | **NA** | **51%** | **NA** | **NA** | **17%** | **85.4% (5)** | **80.2% (5)** | **NA** | **NA** |
| **[59]** | **Keilty** | **2008-2015** | **Canada** | **NA** | **57** | **48 (37-59)** | **416** | **NA** | **NA** | **NA** | **NA** | **25%** | **NA** | **100%** | **22%** | **90.0% (5)** | **77.0% (5)** | **6.4% (5)** | **1.28%** |
| **[60]** | **Simons** | **2008-2017** | **Netherland** | **1** | **81** | **50 (23-81)** | **561** | **95%** | **26%** | **NA** | **NA** | **65%** | **NA** | **NA** | **NA** | **88.4% (5)** | **84.5% (5)** | **2.6% (5)** | **0.52%** |
| **[61]** | **Hong** | **2008-2019** | **China** | **NA** | **61** | **50 (21-82)** | **461** | **93%** | **36%** | **55%** | **NA** | **NA** | **NA** | **NA** | **24%** | **88.2% (5)** | **75.8% (5)** | **NA** | **NA** |
| **[62]** | **Le** | **2009-2012** | **Vietnam** | **1** | **75** | **46 (25-64)** | **126** | **52%** | **36%** | **NA** | **NA** | **2%** | **NA** | **97%** | **25%** | **36.5% (10)** | **29.7% (10)** | **NA** | **NA** |
| **[63]** | **Vieites** | **2009-2012** | **Spain** | **NA** | **60** | **NA** | **267** | **98%** | **26%** | **73%** | **NA** | **NA** | **NA** | **100%** | **NA** | **NA** | **88.5% (5)** | **NA** | **NA** |
| **[64]** | **KimS 2021** | **2009-2013** | **Korea** | **9** | **71** | **44 (20-73)** | **193** | **NA** | **NA** | **NA** | **NA** | **46%** | **NA** | **100%** | **NA** | **81.8% (5)** | **68.6% (5)** | **NA** | **NA** |
| **[65]** | **Cao2019** | **2009-2015** | **China** | **1** | **60** | **NA** | **163** | **90%** | **63%** | **47%** | **NA** | **11%** | **42%** | **77%** | **22%** | **96.2% (5)** | **NA** | **3.9% (5)** | **0.78%** |
| **[66]** | **Akbari** | **2009-2019** | **Iran** | **1** | **120** | **46 (35-57)** | **320** | **NA** | **28%** | **68%** | **NA** | **36%** | **NA** | **NA** | **NA** | **87.0% (5)** | **NA** | **NA** | **NA** |
| **[67]** | **Li2023** | **2009-2020** | **China** | **1** | **33** | **NA** | **123** | **85%** | **46%** | **NA** | **NA** | **3%** | **42%** | **100%** | **20%** | **64.6% (5)** | **43.7% (5)** | **17.9% (5)** | **3.58%** |
| **[68]** | **Su** | **2009-2020** | **China** | **NA** | **46** | **43 (19-71)** | **598** | **NA** | **33%** | **NA** | **NA** | **NA** | **NA** | **NA** | **18%** | **95.5% (5)** | **NA** | **NA** | **NA** |
| **[69]** | **Li** | **2010-2014** | **china** | **1** | **49** | **47 (27-70)** | **139** | **88%** | **NA** | **53%** | **NA** | **NA** | **NA** | **NA** | **18%** | **73.4% (5)** | **65.5% (5)** | **NA** | **NA** |

|  | **Trial/study name** | **Recruitment period** | **country** | **centres** | **Follow-up (Median month** | **Median/Mean age (Range)** | **No. of patients** | **Operability at diagnosis** | **HER2 positive** | **ER positive** | **Treatment type** | **BCS Surgery pts** | **Post-operative chemotherapy recorded** | **Radiotherapy recorded** | **Total pCR** | **Overall Survival**  **(years)** | **Disease-free survival**  **(years)** | **Locoregional recurrence (years)** | Annual Locoregional recurrence% |
| --- | --- | --- | --- | --- | --- | --- | --- | --- | --- | --- | --- | --- | --- | --- | --- | --- | --- | --- | --- |
| **[70]** | **Collins** | **2010-2015** | **Ireland** | **1** | **72** | **50 (39-61)** | **114** | **96%** | **NA** | **97%** | **NA** | **NA** | **NA** | **NA** | **8%** | **80.2% (5)** | **77.7% (5)** | **NA** | **NA** |
| [71] | **Zetterlund** | **2010-2015** | **Swiss** | **16** | **48** | **48 (22-84)** | **417** | **96%** | **32%** | **65%** | **NA** | **28%** | **NA** | **93%** | **NA** | **87.8% (5)** | **NA** | **4.1% (5)** | **0.82%** |
| **[72]** | **Leone** | **2010-2016** | **USA** | **NA** | **35** | **53 (18-90)** | **68065** | **NA** | **35%** | **NA** | **NA** | **36%** | **NA** | **NA** | **26%** | **79% (5)** | **NA** | **NA** | **NA** |
| **[73]** | **Wu 2021** | **2010-2016** | **Korea** | **1** | **63** | **41 (24-61)** | **310** | **NA** | **34%** | **NA** | **NA** | **NA** | **NA** | **44%** | **13%** | **91.3% (5)** | **NA** | **11% (5)** | **2.20%** |
| **[74]** | **Zhang2020** | **2010-2016** | **China** | **1** | **44** | **48 (30-69)** | **114** | **NA** | **NA** | **NA** | **NA** | **NA** | **38%** | **100%** | **NA** | **NA** | **69.3% (5)** | **NA** | **NA** |
| **[75]** | **Wu 2020** | **2010-2016** | **Korea** | **1** | **67** | **NA** | **1395** | **NA** | **36%** | **NA** | **NA** | **NA** | **NA** | **NA** | **NA** | **86.4% (5)** | **78.5% (5)** | **5.5% (5)** | **1.10%** |
| **[76]** | **Wu 2022** | **2010-2016** | **China** | **NA** | **72** | **36 (23-40)** | **375** | **63%** | **NA** | **NA** | **NA** | **NA** | **NA** | **63%** | **12%** | **NA** | **65.3% (5)** | **16.6% (5)** | **3.32%** |
| **[77]** | **Sang** | **2010-2019** | **China** | **1** | **55** | **NA** | **2080** | **NA** | **40%** | **58%** | **NA** | **13%** | **NA** | **NA** | **28%** | **91.8% (5)** | **85.5% (5)** | **6.3% (5)** | **1.26%** |
| **[78]** | **Park** | **2010-2019** | **Korea** | **12** | **75** | **47 (24-84)** | **1273** | **91%** | **27%** | **70%** | **NA** | **67%** | **28%** | **NA** | **NA** | **87.5% (5)** | **71.6% (5)** | **6.6% (5)** | **1.32%** |
| **[79]** | **Wang 2018** | **2011-2013** | **China** | **NA** | **61** | **50 (24-72)** | **217** | **NA** | **11%** | **77%** | **NA** | **0%** | **98%** | **59%** | **NA** | **81.0% (5)** | **73.0% (5)** | **12% (5)** | **2.40%** |
| **[80]** | **De Wild** | **2011-2015** | **Netherlands** | **17** | **70** | **49 (43-57)** | **838** | **NA** | **20%** | **NA** | **NA** | **57%** | **NA** | **NA** | **34%** | **92.2% (5)** | **NA** | **2.2% (5)** | **0.44%** |
| **[81]** | **Li2022** | **2011-2017** | **China** | **1** | **60** | **NA** | **192** | **60%** | **49%** | **74%** | **NA** | **60%** | **NA** | **35%** | **8%** | **61.6% (5)** | **26.7% (5)** | **NA** | **NA** |
| **[82]** | **Suppan** | **2011-2020** | **German** | **1** | **49** | **54 (47-63)** | **367** | **NA** | **35%** | **NA** | **1** | **70%** | **40%** | **NA** | **NA** | **83.0% (5)** | **NA** | **3.0% (5)** | **0.60%** |
| **[83]** | **ZhangS** | **2011-2022** | **China** | **1** | **40** | **NA** | **3070** | **NA** | **0%** | **NA** | **NA** | **12%** | **NA** | **69%** | **15%** | **90.4% (5)** | **82.6% (5)** | **NA** | **NA** |
| **[84]** | **CAO2020** | **2012-2014** | **China** | **1** | **57** | **50 (38-62)** | **906** | **98%** | **28%** | **76%** | **NA** | **NA** | **NA** | **NA** | **NA** | **92.2% (5)** | **89.0% (5)** | **NA** | **NA** |
| [85] | **Hou** | **2012-2015** | **China** | **1** | **58** | **NA** | **749** | **NA** | **34%** | **50%** | **1** | **5%** | **76%** | **51%** | **13%** | **85.1% (5)** | **72.9% (5)** | **NA** | **NA** |
| **[86]** | **Hussein** | **2012-2015** | **Egypt** | **1** | **74** | **NA** | **105** | **88%** | **34%** | **75%** | **NA** | **12%** | **82%** | **81%** | **NA** | **77.5% (7)** | **69.5% (7)** | **NA** | **NA** |
| **[87]** | **Jiang** | **2012-2016** | **China** | **1** | **NA** | **49** | **305** | **98%** | **35%** | **55%** | **NA** | **NA** | **NA** | **NA** | **18%** | **73.3% (5)** | **55.2% (5)** | **NA** | **NA** |
| **[88]** | **Lai** | **2012-2018** | **Canada** | **NA** | **40** | **56 (28-87)** | **458** | **NA** | **42%** | **NA** | **NA** | **32%** | **NA** | **NA** | **32%** | **81.9% (5)** | **76.8% (5)** | **NA** | **NA** |
| **[89]** | **Dai** | **2012-2019** | **China** | **1** | **56** | **60 (21-67)** | **116** | **NA** | **59%** | **NA** | **NA** | **0%** | **NA** | **NA** | **14%** | **97.0% (5)** | **92.5% (5)** | **0.0% (5)** | **0.0%** |
| **[90]** | **Huang2023** | **2013-2016** | **China** | **1** | **80** | **52 (22-88)** | **524** | **NA** | **25%** | **67%** | **NA** | **NA** | **NA** | **NA** | **NA** | **NA** | **83.9% (5)** | **NA** | **NA** |
| **[91]** | **Kang** | **2014-2018** | **Korea** | **1** | **NA** | **46 (22-79)** | **1572** | **96%** | **NA** | **68%** | **NA** | **NA** | **NA** | **NA** | **11%** | **88.1% (5)** | **74.6% (5)** | **NA** | **NA** |

**Clinical trials**

|  | Trial/study name | Recruitment period | country | centres | Follow-up (Median month | Median/Mean age (Range) | No. of patients | Operability at diagnosis | HER2 positive | ER positive | Treatment type | BCS Surgery pts (%) | Post-operative chemotherapy recorded | Radiotherapy recorded | Total pCR | Overall Survival (years) | Disease-free survival  (years) | Locoregional recurrence  (years) | Annual Locoregional recurrence% |
| --- | --- | --- | --- | --- | --- | --- | --- | --- | --- | --- | --- | --- | --- | --- | --- | --- | --- | --- | --- |
| [92] | **Deo** | **1997-2001** | **India** | **NA** | **25** | **49 (22-72)** | **101** | **NA** | **NA** | **NA** | **4** | **NA** | **NA** | **NA** |  | **63% (5)** | **61% (5)** | **2% (5)** | **0.40%** |
| [93] | **EORTC 10994** | **2002-2005** | **NA** | **NA** | **57** | **NA** | **1553** | **89%** | **NA** | **NA** | **NA** | **47%** | **NA** | **NA** | **NA** | **NA** | **NA** | **4.9% (5)** | **0.98%** |
| [94] | **Krug** | **2002-2010** | **German** | **NA** | **52** | **49(21-78)** | **817** | **85%** | **25%** | **66%** | **NA** | **36%** | **100%** | **NA** | **10%** | **NA** | **NA** | **11.4% (5)** | **2.28%** |
| [95] | **INTENS** | **2006-2009** | **Dutch** | **21** | **72** | **49 (24-70)** | **201** | **NA** | **21%** | **NA** | **2** | **NA** | **NA** | **NA** | **24%** | **80% (5)** | **76% (5)** | **NA** | **NA** |
| [96] | **NSABP-B27** | **2008-2015** | **Jordan** | **1** | **NA** | **45 (24-78)** | **121** | **NA** | **100%** | **68%** | **2** | **22%** | **NA** | **NA** | **49%** | **83.4% (5)** | **74.1% (5)** | **NA** | **NA** |
| [97] | **ACOSOG**  **Z1071** | **2009-2011** | **USA** | **NA** | **71** | **NA** | **701** | **95%** | **33%** | **46%** | **5** | **100%** | **NA** | **100%** | **41%** | **84.9% (5)** | **83.7% (5)** | **7.0% (5)** | **1.40%** |
| [97] | **ACOSOG**  **Z1071** | **2009-2011** | **USA** | **NA** | **71** | **NA** | **701** | **95%** | **33%** | **46%** | **5** | **100%** | **NA** | **0%** | **55%** | **89.8% (5)** | **87.3% (5)** | **10.0% (5)** | **2.00%** |
| [97] | **ACOSOG**  **Z1071** | **2009-2011** | **USA** | **NA** | **71** | **NA** | **701** | **95%** | **33%** | **46%** | **5** | **0%** | **NA** | **100%** | **34%** | **84.4% (5)** | **79.3% (5)** | **3.0% (5)** | **0.60%** |
| [97] | **ACOSOG**  **Z1071** | **2009-2011** | **USA** | **NA** | **71** | **NA** | **701** | **95%** | **33%** | **46%** | **5** | **0%** | **NA** | **0%** | **49%** | **82.1% (5)** | **75.3% (5)** | **7.0% (5)** | **1.40%** |
| [98] | **CALGB 40603** | **UN-2020** | **USA** | **NA** | **NA** | **NA** | **443** | **98%** | **1%** | **10%** | **2** | **100%** | **NA** | **0%** | **39%** | **97.2% (5)** | **NA** | **12.1% (5)** | **2.42%** |
| [98] | **CALGB 40603** | **UN-2020** | **USA** | **NA** | **NA** | **NA** | **443** | **98%** | **1%** | **10%** | **2** | **100%** | **NA** | **0%** | **43%** | **98.2% (5)** | **NA** | **12.4% (5)** | **2.48%** |
| [98] | **CALGB 40603** | **UN-2020** | **USA** | **NA** | **NA** | **NA** | **443** | **98%** | **1%** | **10%** | **1** | **100%** | **NA** | **0%** | **49%** | **95.6% (5)** | **NA** | **11.5% (5)** | **2.30%** |
| [98] | **CALGB 40603** | **UN-2020** | **USA** | **NA** | **NA** | **NA** | **443** | **98%** | **1%** | **10%** | **1** | **100%** | **NA** | **0%** | **60%** | **95.6% (5)** | **NA** | **8.0% (5)** | **1.60%** |
| [99] | **Iwase** | **2010-2011** | **Japan** | **10** | **79** | **47 (30-69)** | **154** | **NA** | **NA** | **50%** | **NA** | **NA** | **NA** | **NA** | **24%** | **83.1% (6)** | **74.7% (6.6)** | **NA** | **NA** |
| [100] | **Yang 2023** | **2010-2018** | **China** | **NA** | **72.4** | **48 (27-73)** | **433** | **NA** | **NA** | **NA** | **NA** | **71%** | **NA** | **NA** | **NA** | **94.9 % (5)** | **NA** | **NA** | **NA** |
| [101] | **Train-2** | **2013-2016** | **Netherlands** | **37** | **72** | **NA** | **1124** | **NA** | **NA** | **NA** | **NA** | **54%** | **2%** | **81%** | **49%** | **92.0% (5)** | **NA** | **NA** | **NA** |
| [102] | **Yan** | **2014-2019** | **China** | **1** | **48.2** | **NA** | **200** | **100%** | **NA** | **NA** | **2** | **NA** | **NA** | **NA** | **NA** | **88% (5)** | **85% (5)** | **NA** | **NA** |
| [103] | **SICOG9908** | **1999-2004** | **Italy** | **NA** | **74** | **64 (27-73)** | **200** | **8%** | **22%** | **60%** | **2** | **NA** | **NA** | **NA** | **NA** | **75.5% (5)** | **NA** | **9.5% (5)** | **1.90%** |
| [104] | **UNICANCER-**  **PEGASE 07** | **2001-2005** | **France** | **14** | **60** | **NA** | **174** | **NA** | **34%** | **43%** | **NA** | **NA** | **NA** | **NA** | **20%** | **70.0% (5)** | **55.0% (5)** | **8.0% (5)** | **1.60%** |
| [105] | **NeoSphere** | **2007-2009** | **NA** | **59** | **NA** | **NA** | **417** | **NA** | **100%** | **NA** | **NA** | **NA** | **NA** | **NA** | **NA** | **NA** | **80.1% (5)** | **NA** | **NA** |
| [106] | **ABCSG 29** | **2005-2009** | **German** | **NA** | **55** | **NA** | **693** | **96%** | **17%** | **NA** | **NA** | **NA** | **NA** | **NA** | **NA** | **84.0% (5)** | **70.0% (5)** | **5.8% (5)** | **1.16%** |
| [107] | **Gepar-Quinto** | **2007-2010** | **German** | **NA** | **61** | **NA** | **128** | **NA** | **100%** | **50%** | **NA** | **NA** | **NA** | **NA** | **31%** | **88.5% (5)** | **77.0% (5)** | **NA** | **NA** |
| [108] | **Neo-ALTTO** | **2008-2010** | **NA** | **NA** | **80** | **NA** | **455** | **NA** | **100%** | **NA** | **NA** | **NA** | **NA** | **NA** | **NA** | **82.0% (6)** | **NA** | **5% (6)** | **0.83%** |
| [109] | **CALGB40601** | **2008-2012** | **NA** | **NA** | **84** | **NA** | **569** | **NA** | **100%** | **NA** | **2** | **NA** | **42%** | **NA** | **47%** | **88.5% (5)** | **NA** | **NA** | **NA** |
| [110] | **CHER-Lob** | **2006-2010** | **NA** | **12** | **108** | **NA** | **114** | **NA** | **100%** | **59%** | **NA** | **NA** | **NA** | **NA** | **33%** | **86.0% (9)** | **NA** | **12.0% (9)** | **1.33%** |
| [111] | **Neo-STOP** | **2015-2018** | **USA** | **NA** | **38** | **51 (29-70)** | **101** | **NA** | **NA** | **NA** | **NA** | **49%** | **NA** | **NA** | **54%** | **85.5% (5)** | **NA** | **NA** | **NA** |
| [112] | **NACATRINE** | **2017-2021** | **Brazil** | **1** | **48** | **45** | **146** | **74%** | **NA** | **NA** | **NA** | **32%** | **NA** | **NA** | **37%** | **60.0% (5)** | **NA** | **NA** | **NA** |

**NA-not available, BCS-breast conserving surgery, *Chemotherapy category - 1. containing any platinum (cisplatin or carboplatin); 2. containing taxane (docetaxel or paclitaxel) but not platinum; 3. not containing platinum or taxane but containing anthracycline (epirubicin or doxorubicin); 4. not containing platinum, not containing taxane nor anthracycline (e.g., CMF); 5. not specified**

1. Arun, B., et al., *Response to neoadjuvant systemic therapy for breast cancer in BRCA mutation carriers and noncarriers: a single-institution experience.* J Clin Oncol, 2011. **29**(28): p. 3739-46.

2. Miller, M., et al., *Tumor response ratio predicts overall survival in breast cancer patients treated with neoadjuvant chemotherapy.* Ann Surg Oncol, 2014. **21**(10): p. 3317-23.

3. Meattini, I., et al., *Postmastectomy radiotherapy for locally advanced breast cancer receiving neoadjuvant chemotherapy.* Biomed Res Int, 2014. **2014**: p. 719175.

4. Nakhlis, F., et al., *The Impact of Residual Disease After Preoperative Systemic Therapy on Clinical Outcomes in Patients with Inflammatory Breast Cancer.* Ann Surg Oncol, 2017. **24**(9): p. 2563-2569.

5. Noh, J.M., et al., *Is elective nodal irradiation beneficial in patients with pathologically negative lymph nodes after neoadjuvant chemotherapy and breast-conserving surgery for clinical stage II-III breast cancer? A multicentre retrospective study (KROG 12-05).* Br J Cancer, 2014. **110**(6): p. 1420-6.

6. Spring, L., et al., *Pathologic Complete Response After Neoadjuvant Chemotherapy and Long-Term Outcomes Among Young Women With Breast Cancer.* J Natl Compr Canc Netw, 2017. **15**(10): p. 1216-1223.

7. Huang, L., et al., *Risk factors of locoregional relapse in locally advanced breast cancer treated with neoadjuvant chemotherapy following mastectomy and radiotherapy.* Oncotarget, 2017. **8**(24): p. 39703-39710.

8. Ma, J.C., et al., *The Effect of Postmastectomy Radiotherapy on Breast Cancer Patients After Neoadjuvant Chemotherapy by Molecular Subtype.* Ann Surg Oncol, 2021. **28**(9): p. 5084-5095.

9. Alvarado-Miranda, A., et al., *Concurrent chemo-radiotherapy following neoadjuvant chemotherapy in locally advanced breast cancer.* Radiat Oncol, 2009. **4**: p. 24.

10. Kobayashi, K., et al., *Prognostic significance of histological therapeutic effect in preoperative chemotherapy for breast cancer.* Pathol Int, 2016. **66**(1): p. 8-14.

11. Yang, T.J., et al., *The Effect of Molecular Subtype and Residual Disease on Locoregional Recurrence in Breast Cancer Patients Treated with Neoadjuvant Chemotherapy and Postmastectomy Radiation.* Ann Surg Oncol, 2015. **22 Suppl 3**(Suppl 3): p. S495-501.

12. Chen, S., et al., *Prognostic value of a positive-to-negative change in hormone receptor status after neoadjuvant chemotherapy in patients with hormone receptor-positive breast cancer.* Ann Surg Oncol, 2012. **19**(9): p. 3002-11.

13. Huang, Z., et al., *Postmastectomy Radiation Therapy Based on Pathologic Nodal Status in Clinical Node-Positive Stage II to III Breast Cancer Treated with Neoadjuvant Chemotherapy.* Int J Radiat Oncol Biol Phys, 2020. **108**(4): p. 1030-1039.

14. Terman, E., et al., *The impact of race and age on response to neoadjuvant therapy and long-term outcomes in Black and White women with early-stage breast cancer.* Breast Cancer Res Treat, 2023. **200**(1): p. 75-83.

15. Huang, O., et al., *Retrospective analysis of 119 Chinese noninflammatory locally advanced breast cancer cases treated with intravenous combination of vinorelbine and epirubicin as a neoadjuvant chemotherapy: a median follow-up of 63.4 months.* BMC Cancer, 2009. **9**: p. 375.

16. Lee, N.K., et al., *Stage-to-stage comparison of neoadjuvant chemotherapy versus adjuvant chemotherapy in pathological lymph node positive breast cancer patients.* Jpn J Clin Oncol, 2012. **42**(11): p. 995-1001.

17. Min, S.Y., et al., *Locoregional recurrence of breast cancer in patients treated with breast conservation surgery and radiotherapy following neoadjuvant chemotherapy.* Int J Radiat Oncol Biol Phys, 2011. **81**(5): p. e697-705.

18. Kim, M.M., et al., *Pathologic complete response to neoadjuvant chemotherapy with trastuzumab predicts for improved survival in women with HER2-overexpressing breast cancer.* Ann Oncol, 2013. **24**(8): p. 1999-2004.

19. Noh, J.M., et al., *Prognostic significance of nodal involvement region in clinical stage IIIc breast cancer patients who received primary systemic treatment, surgery, and radiotherapy.* Breast, 2015. **24**(5): p. 637-41.

20. Takada, M., et al., *Prediction of postoperative disease-free survival and brain metastasis for HER2-positive breast cancer patients treated with neoadjuvant chemotherapy plus trastuzumab using a machine learning algorithm.* Breast Cancer Res Treat, 2018. **172**(3): p. 611-618.

21. Garcia-Torralba, E., et al., *A new prognostic model including immune biomarkers, genomic proliferation tumor markers (AURKA and MYBL2) and clinical-pathological features optimizes prognosis in neoadjuvant breast cancer patients.* Front Oncol, 2023. **13**: p. 1182725.

22. Murchison, S., et al., *Locoregional Recurrence and Survival Outcomes in Breast Cancer Treated With Modern Neoadjuvant Chemotherapy: A Contemporary Population-based Analysis.* Clin Breast Cancer, 2022. **22**(7): p. e773-e787.

23. Chang, J.H., et al., *Prognostic Significance of Inner Quadrant Involvement in Breast Cancer Treated with Neoadjuvant Chemotherapy.* J Breast Cancer, 2016. **19**(4): p. 394-401.

24. Jwa, E., et al., *Locoregional Recurrence by Tumor Biology in Breast Cancer Patients after Preoperative Chemotherapy and Breast Conservation Treatment.* Cancer Res Treat, 2016. **48**(4): p. 1363-1372.

25. Wong, S.M., et al., *Prognostic Significance of Residual Axillary Nodal Micrometastases and Isolated Tumor Cells After Neoadjuvant Chemotherapy for Breast Cancer.* Ann Surg Oncol, 2019. **26**(11): p. 3502-3509.

26. Michel, L.L., et al., *Locoregional risk assessment after neoadjuvant chemotherapy in patients with primary breast cancer: clinical utility of the CPS + EG score.* Breast Cancer Res Treat, 2019. **177**(2): p. 437-446.

27. Dredze, L.M., et al., *Neoadjuvant therapy with doxorubicin-cyclophosphamide followed by weekly paclitaxel in early breast cancer: a retrospective analysis of 200 consecutive patients treated in a single center with a median follow-up of 9.5 years.* Breast Cancer Res Treat, 2022. **193**(3): p. 597-612.

28. Chen, X., et al., *Co-mutation of TP53 and PIK3CA in residual disease after neoadjuvant chemotherapy is associated with poor survival in breast cancer.* J Cancer Res Clin Oncol, 2019. **145**(5): p. 1235-1242.

29. Zhang, J., et al., *Impact of the addition of carboplatin to anthracycline-taxane-based neoadjuvant chemotherapy on survival in BRCA1/2-mutated triple-negative breast cancer.* Int J Cancer, 2021. **148**(4): p. 941-949.

30. Kwon, B.R., et al., *Microcalcifications and Peritumoral Edema Predict Survival Outcome in Luminal Breast Cancer Treated with Neoadjuvant Chemotherapy.* Radiology, 2022. **304**(2): p. 310-319.

31. Shin, H.C., et al., *Breast-conserving surgery after tumor downstaging by neoadjuvant chemotherapy is oncologically safe for stage III breast cancer patients.* Ann Surg Oncol, 2013. **20**(8): p. 2582-9.

32. Kim, S.I., et al., *Molecular subtypes and tumor response to neoadjuvant chemotherapy in patients with locally advanced breast cancer.* Oncology, 2010. **79**(5-6): p. 324-30.

33. Jimbo, K., et al., *Oncological safety of breast-conserving surgery after primary systemic chemotherapy in cT3-4 breast cancer patients.* Surg Today, 2015. **45**(10): p. 1255-62.

34. Ohri, N., et al., *Postmastectomy Radiation in Breast Cancer Patients With Pathologically Positive Lymph Nodes After Neoadjuvant Chemotherapy: Usage Rates and Survival Trends.* Int J Radiat Oncol Biol Phys, 2017. **99**(3): p. 549-559.

35. Haque, W., et al., *Response rates and pathologic complete response by breast cancer molecular subtype following neoadjuvant chemotherapy.* Breast Cancer Res Treat, 2018. **170**(3): p. 559-567.

36. Prakash, I., et al., *Time to surgery among women treated with neoadjuvant systemic therapy and upfront surgery for breast cancer.* Breast Cancer Res Treat, 2021. **186**(2): p. 535-550.

37. van Nijnatten, T.J., et al., *Prognosis of residual axillary disease after neoadjuvant chemotherapy in clinically node-positive breast cancer patients: isolated tumor cells and micrometastases carry a better prognosis than macrometastases.* Breast Cancer Res Treat, 2017. **163**(1): p. 159-166.

38. LeVasseur, N., et al., *Impact of pathologic complete response on survival after neoadjuvant chemotherapy in early-stage breast cancer: a population-based analysis.* J Cancer Res Clin Oncol, 2020. **146**(2): p. 529-536.

39. Swisher, S.K., et al., *Locoregional Control According to Breast Cancer Subtype and Response to Neoadjuvant Chemotherapy in Breast Cancer Patients Undergoing Breast-conserving Therapy.* Ann Surg Oncol, 2016. **23**(3): p. 749-56.

40. Carrara, G.F., et al., *Breast-conserving surgery in locally advanced breast cancer submitted to neoadjuvant chemotherapy. Safety and effectiveness based on ipsilateral breast tumor recurrence and long-term follow-up.* Clinics (Sao Paulo), 2017. **72**(3): p. 134-142.

41. Nakajima, N., et al., *Clinical outcomes and prognostic factors in patients with stage II-III breast cancer treated with neoadjuvant chemotherapy followed by surgery and postmastectomy radiation therapy in the modern treatment era.* Adv Radiat Oncol, 2018. **3**(3): p. 271-279.

42. Luo, J., et al., *Internal Mammary Node Irradiation (IMNI) Improves Survival Outcome for Patients With Clinical Stage II-III Breast Cancer After Preoperative Systemic Therapy.* Int J Radiat Oncol Biol Phys, 2019. **103**(4): p. 895-904.

43. Al-Tweigeri, T., et al., *Impact of Pathologic Complete Response following Neoadjuvant Chemotherapy +/- Trastuzumab in Locally Advanced Breast Cancer.* J Oncol, 2021. **2021**: p. 6639763.

44. Antolin, S., et al., *Primary systemic therapy in HER2-positive operable breast cancer using trastuzumab and chemotherapy: efficacy data, cardiotoxicity and long-term follow-up in 142 patients diagnosed from 2005 to 2016 at a single institution.* Breast Cancer (Dove Med Press), 2019. **11**: p. 29-42.

45. Chen, S.C., et al., *Discrepancy of Breast and Axillary Pathologic Complete Response and Outcomes in Different Subtypes of Node-positive Breast Cancer after Neoadjuvant Chemotherapy.* J Cancer, 2021. **12**(17): p. 5365-5374.

46. Hannikainen, E.N., J. Mattson, and P. Karihtala, *Predictors of successful neoadjuvant treatment in HER2‑positive breast cancer.* Oncol Lett, 2023. **26**(4): p. 434.

47. Fujita, N., et al., *Response-Guided Omission of Anthracycline in Patients with HER2-Positive Early Breast Cancer Treated with Neoadjuvant Taxane and Trastuzumab: 5-Year Follow-Up of Prognostic Study Using Propensity Score Matching.* Oncology, 2022. **100**(5): p. 257-266.

48. Xin, F., et al., *Number of negative lymph nodes as a prognostic factor for ypN0-N1 breast cancer patients undergoing neoadjuvant chemotherapy.* Tumour Biol, 2016. **37**(6): p. 8445-54.

49. Kim, H., et al., *Sentinel Lymph Node Biopsy in Breast Cancer Patients With Pathological Complete Response in the Axillary Lymph Node After Neoadjuvant Chemotherapy.* J Breast Cancer, 2021. **24**(6): p. 531-541.

50. Luangdilok, S., N. Samarnthai, and K. Korphaisarn, *Association between Pathological Complete Response and Outcome Following Neoadjuvant Chemotherapy in Locally Advanced Breast Cancer Patients.* J Breast Cancer, 2014. **17**(4): p. 376-85.

51. Barranger, E., et al., *Effect of Neoadjuvant Chemotherapy on the Surgical Treatment of Patients With Locally Advanced Breast Cancer Requiring Initial Mastectomy.* Clin Breast Cancer, 2015. **15**(5): p. e231-5.

52. Zhu, X., et al., *Neoadjuvant Chemotherapy Plays an Adverse Role in the Prognosis of Grade 2 Breast Cancer.* J Cancer, 2019. **10**(23): p. 5661-5670.

53. Zhang, Y., et al., *Impact of Postmastectomy Radiotherapy on Locoregional Control and Disease-Free Survival in Patients with Breast Cancer Treated with Neoadjuvant Chemotherapy.* J Oncol, 2021. **2021**: p. 6632635.

54. Munoz-Montano, W., et al., *Prognostic Value of the Pretreatment Neutrophil-to-Lymphocyte Ratio in Different Phenotypes of Locally Advanced Breast Cancer During Neoadjuvant Systemic Treatment.* Clin Breast Cancer, 2020. **20**(4): p. 307-316 e1.

55. Asaoka, M., et al., *Clinical and pathological predictors of recurrence in breast cancer patients achieving pathological complete response to neoadjuvant chemotherapy.* Eur J Surg Oncol, 2019. **45**(12): p. 2289-2294.

56. Woo, J., et al., *Breast radiologic complete response is associated with favorable survival outcomes after neoadjuvant chemotherapy in breast cancer.* Eur J Surg Oncol, 2021. **47**(2): p. 232-239.

57. Chun, J.W., et al., *Comparison of survival outcomes for axillary surgery extent based on intraoperative sentinel lymph node biopsy result after neoadjuvant chemotherapy for breast cancer.* Breast Cancer Res Treat, 2021. **187**(3): p. 647-655.

58. Gwark, S., et al., *Survival After Breast-Conserving Surgery Compared with that After Mastectomy in Breast Cancer Patients Receiving Neoadjuvant Chemotherapy.* Ann Surg Oncol, 2023. **30**(5): p. 2845-2853.

59. Keilty, D., et al., *Patterns of Recurrence and Predictors of Survival in Breast Cancer Patients Treated with Neoadjuvant Chemotherapy, Surgery, and Radiation.* Int J Radiat Oncol Biol Phys, 2020. **108**(3): p. 676-685.

60. Simons, J.M., et al., *Disease-free and overall survival after neoadjuvant chemotherapy in breast cancer: breast-conserving surgery compared to mastectomy in a large single-centre cohort study.* Breast Cancer Res Treat, 2021. **185**(2): p. 441-451.

61. Hong, J., et al., *Association of tumor-infiltrating lymphocytes before and after neoadjuvant chemotherapy with pathological complete response and prognosis in patients with breast cancer.* Cancer Med, 2021. **10**(22): p. 7921-7933.

62. Le, D.T., et al., *Neoadjuvant Doxorubicin-Paclitaxel Combined Chemotherapy in Patients with Inoperable Stage III Breast Cancer: A Retrospective Cohort Study with 10 Years of Follow-Up in Vietnam.* Oncol Ther, 2023. **11**(3): p. 327-341.

63. Vieites, B., et al., *Predictive and prognostic value of total tumor load in sentinel lymph nodes in breast cancer patients after neoadjuvant treatment using one-step nucleic acid amplification: the NEOVATTL study.* Clin Transl Oncol, 2021. **23**(7): p. 1377-1385.

64. Kim, H., et al., *Outcome of radiotherapy for clinically overt metastasis to the internal mammary lymph node in patients receiving neoadjuvant chemotherapy and breast cancer surgery.* Breast, 2021. **55**: p. 112-118.

65. Cao, L., et al., *The Role of the Neo-Bioscore Staging System in Guiding the Optimal Strategies for Regional Nodal Irradiation Following Neoadjuvant Treatment in Breast Cancer Patients with cN1 and ypN0-1.* Ann Surg Oncol, 2019. **26**(2): p. 343-355.

66. Akbari, M.E., et al., *Neoadjuvant VS adjuvant chemotherapy in patients with locally advanced breast cancer; a retrospective cohort study.* Ann Med Surg (Lond), 2022. **84**: p. 104921.

67. Li, S., et al., *Nodal response to primary systemic therapy predicts prognosis of cN3c breast cancer patients receiving multimodality therapy.* Breast, 2023. **70**: p. 92-99.

68. Su, A., et al., *Impact of Atypical Hyperplasia at Surgical Margins on breast cancer outcomes in patients treated with neoadjuvant chemotherapy.* Front Oncol, 2023. **13**: p. 1202689.

69. Li, S., et al., *Comparison of the efficacy and survival analysis of neoadjuvant chemotherapy for Her-2-positive breast cancer.* Drug Des Devel Ther, 2018. **12**: p. 3085-3093.

70. Collins, P.M., et al., *Neoadjuvant chemotherapy for luminal a breast cancer: Factors predictive of histopathologic response and oncologic outcome.* Am J Surg, 2021. **222**(2): p. 368-376.

71. Zetterlund, L., et al., *Long-term prognosis in breast cancer is associated with residual disease after neoadjuvant systemic therapy but not with initial nodal status.* Br J Surg, 2021. **108**(5): p. 583-589.

72. Leone, J.P., et al., *Efficacy of neoadjuvant chemotherapy in male breast cancer compared with female breast cancer.* Cancer, 2022. **128**(21): p. 3796-3803.

73. Wu, Z.Y., et al., *Oncologic Outcomes of Nipple-sparing Mastectomy and Immediate Reconstruction After Neoadjuvant Chemotherapy for Breast Cancer.* Ann Surg, 2021. **274**(6): p. e1196-e1201.

74. Zhang, L., et al., *The Impact of Preoperative Radiomics Signature on the Survival of Breast Cancer Patients With Residual Tumors After NAC.* Front Oncol, 2020. **10**: p. 523327.

75. Wu, Z.Y., et al., *Long-term Oncologic Outcomes of Immediate Breast Reconstruction vs Conventional Mastectomy Alone for Breast Cancer in the Setting of Neoadjuvant Chemotherapy.* JAMA Surg, 2020. **155**(12): p. 1142-1150.

76. Wu, Z.Y., et al., *Oncologic outcomes of immediate breast reconstruction in young women with breast cancer receiving neoadjuvant chemotherapy.* Breast Cancer Res Treat, 2022. **191**(2): p. 345-354.

77. Sang, Y., et al., *Surgical options of the breast and clinical outcomes of breast cancer patients after neoadjuvant chemotherapy: A single-center retrospective study.* Front Oncol, 2022. **12**: p. 984587.

78. Park, Y., et al., *Omission of axillary lymph node dissection in patients with ypN+ breast cancer after neoadjuvant chemotherapy: A retrospective multicenter study (KROG 21-06).* Eur J Surg Oncol, 2023. **49**(3): p. 589-596.

79. Wang, X., et al., *Locoregional recurrence-associated factors and risk-adapted postmastectomy radiotherapy for breast cancer staged in cT1-2N0-1 after neoadjuvant chemotherapy.* Cancer Manag Res, 2018. **10**: p. 4105-4112.

80. de Wild, S.R., et al., *De-escalation of radiotherapy after primary chemotherapy in cT1-2N1 breast cancer (RAPCHEM; BOOG 2010-03): 5-year follow-up results of a Dutch, prospective, registry study.* Lancet Oncol, 2022. **23**(9): p. 1201-1210.

81. Li, C.L., et al., *The impact of age group in breast cancer survival outcome according to neoadjuvant treatment response: A matched case-control study.* Kaohsiung J Med Sci, 2022. **38**(3): p. 277-282.

82. Suppan, C., et al., *Patterns of Recurrence after Neoadjuvant Therapy in Early Breast Cancer, according to the Residual Cancer Burden Index and Reductions in Neoadjuvant Treatment Intensity.* Cancers (Basel), 2021. **13**(10).

83. Zhang, S., et al., *Prognoses of Patients with Hormone Receptor-Positive and Human Epidermal Growth Factor Receptor 2-Negative Breast Cancer Receiving Neoadjuvant Chemotherapy before Surgery: A Retrospective Analysis.* Cancers (Basel), 2023. **15**(4).

84. Cao, X., et al., *Combination of preoperative fibrinogen concentration and neutrophil-to-lymphocyte ratio for prediction of the prognosis of patients with resectable breast cancer.* Oncol Lett, 2020. **20**(5): p. 200.

85. Hou, N., et al., *Development, verification, and comparison of a risk stratification model integrating residual cancer burden to predict individual prognosis in early-stage breast cancer treated with neoadjuvant therapy.* ESMO Open, 2021. **6**(5): p. 100269.

86. Hussein, M.A., et al., *Expression of PD-L1 in Locally Advanced Breast Cancer and Its Impact on Neoadjuvant Chemotherapy Response.* Asian Pac J Cancer Prev, 2022. **23**(6): p. 2095-2103.

87. Jiang, C., et al., *Prognostic value of a modified systemic inflammation score in breast cancer patients who underwent neoadjuvant chemotherapy.* BMC Cancer, 2022. **22**(1): p. 1249.

88. Lai, V., et al., *Impact of wait time from neoadjuvant chemotherapy to surgery in breast cancer: Does time to surgery affect patient outcomes? : Time from Neoadjuvant Chemotherapy to Surgery.* Breast Cancer Res Treat, 2020. **184**(3): p. 755-762.

89. Dai, Y., et al., *The impact of postmastectomy radiotherapy on cT1-2N1 breast cancer patients with ypN0 after neoadjuvant chemotherapy: a retrospective study based on real-world data.* Discov Oncol, 2023. **14**(1): p. 2.

90. Huang, K., et al., *Effect of preoperative peripheral blood platelet volume index on prognosis in patients with invasive breast cancer.* Future Oncol, 2023.

91. Kang, S., et al., *Pathological complete response, long-term outcomes, and recurrence patterns in HER2-low versus HER2-zero breast cancer after neoadjuvant chemotherapy.* Eur J Cancer, 2022. **176**: p. 30-40.

92. Deo, S.V., et al., *Randomized trial comparing neo-adjuvant versus adjuvant chemotherapy in operable locally advanced breast cancer (T4b N0-2 M0).* J Surg Oncol, 2003. **84**(4): p. 192-7.

93. Gillon, P., et al., *Factors predictive of locoregional recurrence following neoadjuvant chemotherapy in patients with large operable or locally advanced breast cancer: An analysis of the EORTC 10994/BIG 1-00 study.* Eur J Cancer, 2017. **79**: p. 226-234.

94. Krug, D., et al., *Post-Mastectomy Radiotherapy After Neoadjuvant Chemotherapy in Breast Cancer: A Pooled Retrospective Analysis of Three Prospective Randomized Trials.* Ann Surg Oncol, 2019. **26**(12): p. 3892-3901.

95. Vriens, B., et al., *Improved survival for sequentially as opposed to concurrently delivered neoadjuvant chemotherapy in non-metastatic breast cancer.* Breast Cancer Res Treat, 2017. **165**(3): p. 593-600.

96. Abdel-Razeq, H., et al., *Four cycles of adriamycin and cyclophosphamide followed by four cycles of docetaxel (NSABP-B27) with concomitant trastuzumab as neoadjuvant therapy for high-risk, early-stage, HER2-positive breast cancer patients.* Onco Targets Ther, 2018. **11**: p. 2091-2096.

97. Haffty, B.G., et al., *Impact of Radiation on Locoregional Control in Women with Node-Positive Breast Cancer Treated with Neoadjuvant Chemotherapy and Axillary Lymph Node Dissection: Results from ACOSOG Z1071 Clinical Trial.* Int J Radiat Oncol Biol Phys, 2019. **105**(1): p. 174-182.

98. Shepherd, J.H., et al., *CALGB 40603 (Alliance): Long-Term Outcomes and Genomic Correlates of Response and Survival After Neoadjuvant Chemotherapy With or Without Carboplatin and Bevacizumab in Triple-Negative Breast Cancer.* J Clin Oncol, 2022. **40**(12): p. 1323-1334.

99. Iwase, M., et al., *Long-term survival analysis of addition of carboplatin to neoadjuvant chemotherapy in HER2-negative breast cancer.* Breast Cancer Res Treat, 2020. **180**(3): p. 687-694.

100. Yang, Y., et al., *Phase III study of HR-positive/HER2-negative/lymph node-positive breast cancer non-responsive to primary chemotherapy: a randomized trial.* NPJ Breast Cancer, 2023. **9**(1): p. 54.

101. van der Voort, A., et al., *Efficacy of neoadjuvant treatment with or without pertuzumab in patients with stage II and III HER2-positive breast cancer: a nationwide cohort analysis of pathologic response and 5-year survival.* Breast, 2022. **65**: p. 110-115.

102. Yan, W., et al., *Lobaplatin-based neoadjuvant chemotherapy for triple-negative breast cancer: a 5-year follow-up of a randomized, open-label, phase II trial.* Ther Adv Med Oncol, 2022. **14**: p. 17588359221107111.

103. Frasci, G., et al., *Preoperative weekly cisplatin, epirubicin, and paclitaxel (PET) improves prognosis in locally advanced breast cancer patients: an update of the Southern Italy Cooperative Oncology Group (SICOG) randomised trial 9908.* Ann Oncol, 2010. **21**(4): p. 707-716.

104. Goncalves, A., et al., *UNICANCER-PEGASE 07 study: a randomized phase III trial evaluating postoperative docetaxel-5FU regimen after neoadjuvant dose-intense chemotherapy for treatment of inflammatory breast cancer.* Ann Oncol, 2015. **26**(8): p. 1692-7.

105. Gianni, L., et al., *5-year analysis of neoadjuvant pertuzumab and trastuzumab in patients with locally advanced, inflammatory, or early-stage HER2-positive breast cancer (NeoSphere): a multicentre, open-label, phase 2 randomised trial.* Lancet Oncol, 2016. **17**(6): p. 791-800.

106. von Minckwitz, G., et al., *Zoledronate for patients with invasive residual disease after anthracyclines-taxane-based chemotherapy for early breast cancer - The Phase III NeoAdjuvant Trial Add-oN (NaTaN) study (GBG 36/ABCSG 29).* Eur J Cancer, 2016. **64**: p. 12-21.

107. Banys-Paluchowski, M., et al., *Clinical Relevance of Collagen Protein Degradation Markers C3M and C4M in the Serum of Breast Cancer Patients Treated with Neoadjuvant Therapy in the GeparQuinto Trial.* Cancers (Basel), 2019. **11**(8).

108. Huober, J., et al., *Survival outcomes of the NeoALTTO study (BIG 1-06): updated results of a randomised multicenter phase III neoadjuvant clinical trial in patients with HER2-positive primary breast cancer.* Eur J Cancer, 2019. **118**: p. 169-177.

109. Fernandez-Martinez, A., et al., *Survival, Pathologic Response, and Genomics in CALGB 40601 (Alliance), a Neoadjuvant Phase III Trial of Paclitaxel-Trastuzumab With or Without Lapatinib in HER2-Positive Breast Cancer.* J Clin Oncol, 2020. **38**(35): p. 4184-4193.

110. Guarneri, V., et al., *Trastuzumab-lapatinib as neoadjuvant therapy for HER2-positive early breast cancer: Survival analyses of the CHER-Lob trial.* Eur J Cancer, 2021. **153**: p. 133-141.

111. Sharma, P., et al., *Randomized Phase II Trial of Anthracycline-free and Anthracycline-containing Neoadjuvant Carboplatin Chemotherapy Regimens in Stage I-III Triple-negative Breast Cancer (NeoSTOP).* Clin Cancer Res, 2021. **27**(4): p. 975-982.

112. de Padua Souza, C., et al., *Neoadjuvant carboplatin in triple-negative breast cancer: results from NACATRINE, a randomized phase II clinical trial.* Breast Cancer Res Treat, 2023.
